# Pup Mortality in a Rapidly Declining Harbour Seal (*Phoca vitulina*) Population

**DOI:** 10.1371/journal.pone.0080727

**Published:** 2013-11-27

**Authors:** Nora Hanson, Dave Thompson, Callan Duck, Simon Moss, Mike Lonergan

**Affiliations:** Sea Mammal Research Unit, Scottish Oceans Institute, University of St Andrews, Fife, United Kingdom; Texas A&M University-Corpus Christi, United States of America

## Abstract

The harbour seal population in Orkney, off the north coast of Scotland, has reduced by 65% between 2001 and 2010. The cause(s) of this decline are unknown but must affect the demographic parameters of the population. Here, satellite telemetry data were used to test the hypothesis that increased pup mortality could be a primary driver of the decline in Orkney. Pup mortality and tag failure parameters were estimated from the duration of operation of satellite tags deployed on harbour seal pups from the Orkney population (*n* = 24) and from another population on the west coast of Scotland (*n* = 24) where abundance was stable. Survival probabilities from both populations were best represented by a common gamma distribution and were not different from one another, suggesting that increased pup mortality is unlikely to be the primary agent in the Orkney population decline. The estimated probability of surviving to 6 months was 0.390 (95% CI 0.297 – 0.648) and tag failure was represented by a Gaussian distribution, with estimated mean 270 (95% CI = 198 – 288) and s.d. 21 (95% CI = 1 – 66) days. These results suggest that adult survival is the most likely proximate cause of the decline. They also demonstrate a novel technique for attaining age-specific mortality rates from telemetry data.

## Introduction

The monitoring of species abundance and distribution, supported by knowledge of population dynamics, provides information critical to wildlife conservation and management. It is also becoming a legislative requirement (e.g. UK Wildlife and Countryside Act 1981, EU Habitats Directive, USA Endangered Species Act 1973, Marine Mammal Act 1972, Canadian Environmental Assessment Act 1992 [1]) and long-term population monitoring efforts have been established for several species to detect changes in their status [2]. As long-lived top predators with typically wide geographical dispersal, marine mammals are often proposed as important indicators of the state of relatively inaccessible marine ecosystems [3], [4]. Perhaps unsurprisingly, it is populations in rapid and/or sustained decline that are of greatest interest because these patterns may be a harbinger of large-scale shifts in marine conditions. However, understanding the proximate and ultimate drivers for population decline has proven challenging for wild populations [5]–[7].

Harbour seals (*Phoca vitulina vitulina*) have been abundant in the north of Scotland, which contained nearly 20% of all European harbour seals in the late 20^th^ century [8]. These populations escaped the worst of the epidemics of Phocine Distemper Virus that caused mass mortalities in most other European populations in 1998 and 2002 [9]. Until recently, harbour seals in Orkney off the north coast of Scotland accounted for nearly half of all British harbour seals. However, aerial surveys detected rapid declines in the Orkney population, as well as in other populations in the northern isles and on the east coast of Scotland, since the late 1990s [10]. Related research has demonstrated that the proportion of animals hauled out, and available to be counted, in Orkney during the aerial survey period is both high and similar to those reported from other areas [11], confirming that the reductions in the counts indicate real declines in abundance rather than changes in seal behaviour. The mean annual rate of decline for Orkney harbour seals has been established at 13% per annum (95% CI: 10.8 – 14.8) over the period 2001–2010 [11].

There are several potential explanations for the declines. Food shortage, inter-specific competition, disease, predation, pollution, deliberate killing or other anthropogenic factors could all impact on harbour seal population abundance [6], [12], [13]. These might affect a combination of emigration, adult mortality, juvenile mortality, and fecundity. Emigration from the Orkney region, or a reduction in immigration to it, seems an unlikely explanation for the decline since harbour seals generally show high site fidelity [14], [15] and none of the neighbouring populations show any signs of having absorbed the large numbers of animals involved [16].This leaves adult and/or juvenile mortality, and fecundity as potential proximate causes of the decline.

Assessing these parameters in wild populations is difficult and often costly [17]. Due to a lack of information on the age structure of populations, mortality rates often are modeled as being constant across several age-classes, although this assumption is widely accepted to be unrealistic [5]. Many empirically derived mammalian survivorship curves show three major components: an early, juvenile phase where initial mortality is high but survival increases with age; an ongoing, or constant, risk of mortality during maturity; and a later phase of increasing mortality risk due to senescence [5], [7], [17], [18]. At present, there are insufficient data available to reliably estimate adult survival and fecundity rates for harbour seals in the UK. The present study investigates pup mortality rates in the first months of life using satellite telemetry.

Harbour seal pups are born in early summer in the UK and weaned after approximately 1 month [6], [19]. The pups are born below the high water mark on intertidal rocks and sand banks and are capable of swimming and diving shortly after birth, although their swimming abilities continue to develop throughout lactation and post-weaning [20]–[22]. Because of this precociousness, it is challenging to determine neonatal mortality rates for harbour seals. As with many other species, the younger age-classes tend to be at a higher risk of predation and disease than older animals [12]. After weaning, harbour seal pup mortality increases over the winter months and is related to the autumn body mass. Pups that are larger in the autumn are more likely to survive through their first year than are smaller pups [23]. Any form of food limitation, in terms of quality, quantity or accessibility throughout lactation and the post-weaning period may have profound effects on pup survival rates directly by affecting pup condition or indirectly by affecting maternal condition and hence provisioning during the lactation period [24], [25]. Given the potential susceptibility of harbour seal pups to multiple mortality risks, we reasoned that high mortality rates in this age-class could be a major contributor to the decline of harbour seals in Orkney.

This study utilises information obtained via satellite telemetry to compare harbour seal pup mortality rates between two populations with contrasting population trends: the Orkney population, which is in decline and another population on the west coast of Scotland at Lismore, which has remained stable over the last ten years [10]. While restricted to the early post-weaning period, the results of this study demonstrate that such data provide useful insights into age-structured population dynamics of harbour seals.

## Methods

### Ethics statement

In the United Kingdom, all harbour seals are protected under the Conservation of Seals Act (1970) and the Marine (Scotland) Act (2010). Accordingly, all seal pup capture and handling procedures were performed under the terms of the UK Home Office licence (60/3303) granted to the Sea Mammal Research Unit (University of St Andrews) and in strict compliance with the Animals (Scientific Procedures) Act 1986. Access to the field sites was permitted by the public access rights in Scotland (Land Reform (Scotland) act of 2003); accordingly, no specific permissions were required to access these locations.

### Capture and tagging

Small, location-only ARGOS satellite transmitters (SPOT tags, Wildlife Computers, Redmond, WA, USA, weight = 50 g) were deployed on 50 female harbour seal pups: 25 between the 23^rd^ – 26^th^ of June 2007 around Lismore (56.476°N, 5.516°W) and 25 between 2^nd^ – 6^th^ of July 2007 in Orkney (59.2°N, 2.6°W). The seals were captured on land and manually restrained while the satellite transmitters were glued to the fur in the mid-dorsal region using quick-setting epoxy. Only very young female pups were tagged. The presence or absence of umbilicus remnants was recorded to provide a general estimate of the time since birth. The length, weight and axial girth of each individual were recorded. Tagging was performed as quickly as possible to minimise disturbance to the animals and disruption of the maternal bond.

### Data analysis

Once deployed, there are three processes that lead to telemetry systems ceasing to produce useful information: the tag can fail because of mechanical faults such as battery or aerial failure, leading to signal loss; the tag can detach; or the animal can die [26]. In the latter two cases, the tag may continue to transmit, though the pattern of transmissions and movement is unlikely to mimic that of a living seal. The location data was examined by one of the authors (D. Thompson) to determine the last date of transmission by each tag while it was attached to a living seal. This subjective decision was based on the pattern of movement of the tags over the last 5 to 10 days during which transmissions were received by the ARGOS. Table 1 contains the date of tag attachment and date of last ‘live’ transmission, along with morphometric data, for each individual.

**Table 1:** Phoca vitulina.

Region	Tag date	Last live	Mass (kg)	Length (cm)	Girth (cm)	Umbilicus
Orkney	03-Jul-07	21-Nov-07	22.6	92	67	0
Orkney	03-Jul-07	05-Nov-07	17.4	83	68	0
Orkney	03-Jul-07	26-Sep-07	11.2	82	50	1
Orkney	06-Jul-07	01-Jan-08	21	93	72	0
Orkney	04-Jul-07	10-Mar-08	20.7	93	67	NA
Orkney	04-Jul-07	06-Aug-07	18	88	68	0
Orkney	04-Jul-07	30-Nov-07	18.2	76	64	0
Orkney	04-Jul-07	01-Dec-07	25.4	89	77	NA
Orkney	04-Jul-07	20-Oct-07	15.6	82	58	0
Orkney	03-Jul-07	11-Apr-08	21	90	73	0
Orkney	02-Jul-07	02-Oct-07	18.6	86	66	0
Orkney	03-Jul-07	27-Aug-07	16.4	80	64	0
Orkney	05-Jul-07	11-Nov-07	19.4	88	68	0
Orkney	06-Jul-07	24-Jul-07	14.2	89	54	0
Orkney	05-Jul-07	21-Dec-07	18.2	87	65	0
Orkney	06-Jul-07	27-Aug-07	21.4	96	66	0
Orkney	04-Jul-07	NA	17.7	85	68	0
Orkney	04-Jul-07	11-Apr-08	20	88	68	0
Orkney	06-Jul-07	22-Jul-07	16.2	85	62	0
Orkney	03-Jul-07	08-Oct-07	12.4	83	57	1
Orkney	06-Jul-07	14-Sep-07	15.8	89	62	0
Orkney	03-Jul-07	16-Sep-07	19.2	85	66	0
Orkney	04-Jul-07	13-Apr-08	23	85	66	0
Orkney	04-Jul-07	29-Feb-08	15	79	60	0
Orkney	04-Jul-07	02-Mar-08	23.6	92	69	0
Lismore	24-Jun-07	28-Jul-07	9.6	70.2	46	1
Lismore	24-Jun-07	04-Jan-08	11.4	84	53	1
Lismore	24-Jun-07	06-Feb-08	13.4	83	51	0
Lismore	25-Jun-07	11-Feb-08	13.2	84	54	1
Lismore	23-Jun-07	16-Jan-08	10.2	80	51	1
Lismore	26-Jun-07	13-Apr-08	11.7	77	53	0
Lismore	23-Jun-07	22-Jan-08	11.2	84	51	0
Lismore	24-Jun-07	19-Nov-07	13.2	79	62	0
Lismore	25-Jun-07	03-Aug-07	12.2	80	54	0
Lismore	24-Jun-07	08-Mar-08	12.4	82	55	0
Lismore	25-Jun-07	25-Nov-07	9.7	82	47	1
Lismore	23-Jun-07	12-Mar-08	10.2	76	50	1
Lismore	24-Jun-07	05-Aug-07	10.2	79	49	0
Lismore	25-Jun-07	01-Oct-07	10.7	75	45	1
Lismore	25-Jun-07	13-Oct-07	10.2	80	50	1
Lismore	25-Jun-07	16-Dec-07	11.2	80	54	1
Lismore	25-Jun-07	25-Feb-08	13.2	84	55.5	1
Lismore	26-Jun-07	08-Jan-08	10.4	75	50	1
Lismore	26-Jun-07	28-Oct-07	11.2	86	51	1
Lismore	26-Jun-07	03-Dec-07	11.6	81	56	0
Lismore	26-Jun-07	15-Nov-07	13.2	77	54	0
Lismore	26-Jun-07	29-Dec-07	13.6	84	49	1
Lismore	26-Jun-07	26-Aug-07	10.7	75	52	0
Lismore	27-Jun-07	NA	12.2	82	57	1
Lismore	26-Jun-07	15-Dec-07	11	81	52	1

Details of harbour seal pups tagged on the north (Orkney) and west (Lismore) coasts of Scotland.

All statistical analyses were performed in R [27]; the data (Appendix S1) used and R files (Appendix S2) of the analyses can be found in the Supplementary Information. The distribution of the overall tag duration (i.e. last ‘live’ transmission date – tag attachment date) was compared between the two populations using Mann-Whitney U tests. The Kolmogorov-Smirnov test first was used to check the assumption that the two samples came from similar distributions. As the distributions looked similar, the p-value was re-estimated by comparison to a null distribution generated by permuting individuals between groups. The sensitivity of the Mann-Whitney U test was investigated by adding a constant to all the dates from Lismore and retesting. The window of values of this constant for which the results of the Mann-Whitney U test were non-significant at the 5% level was identified.

Ultimately, we were interested in assessing the probability that an individual pup would survive to a given time, and if the parameters of pup survival differed between a population in rapid decline and a stable one. However, the likelihood of the observed telemetry data had two major components: the probability of tag survival and the probability of animal survival. ‘System failure’ – or a loss of useful information from the tags – occurred whenever either of these components failed. The SPOT tags were expected to exhaust their batteries in late February, based on their configuration and an assumed pattern of diving and hauling out. Mechanical faults, such as detachment from the animal, failure of software or electronic components and damage to the antenna, can also end tags' operation [26]. When tag failure occurs prior to animal death, the data are considered to be ‘right-censored’ [7]. Importantly, in the present study right-censorship could not be determined because pup death was never observed directly: it could have occurred at any time after the date of the last ‘live’ transmission. Additionally, pups were tagged as young as possible and always prior to weaning but the exact number of days since their birth was unknown, giving ‘left-truncated’ data. We assumed that survival time and censoring time were independent, random variables. A model describing the probability that the last ‘live’ transmission, from a pup born on day *b* and tagged on day *d*, occurs on day *x* (where *x>d>b*) was defined as:
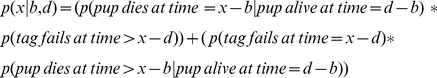
(1)


Giving:
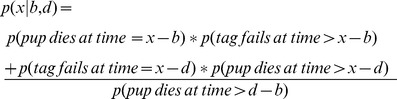
(2)


**Figure 1:**
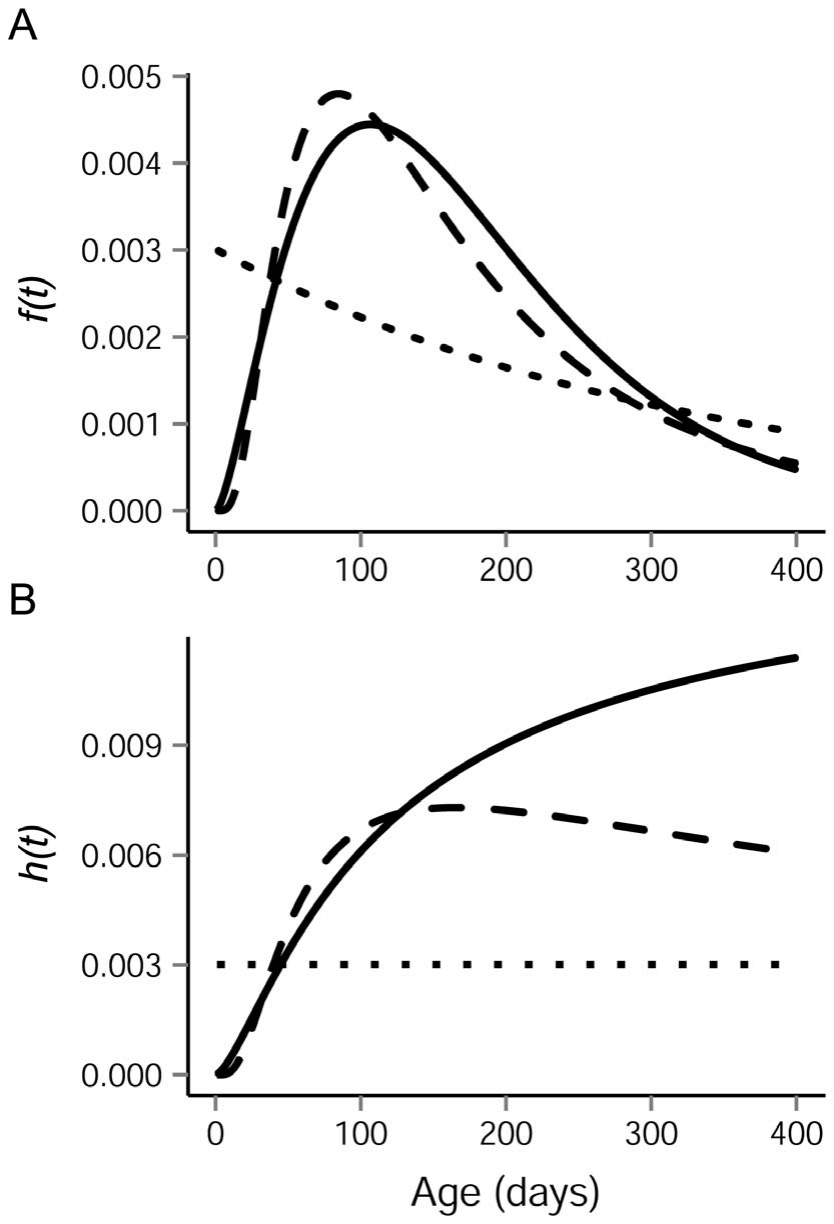
Harbour seal pup mortality and hazard rates. (A) The probability density function of mortality events (*f(t)*) and (B) the hazard rate (*h(t)*) plotted against age for exponential (dotted lines), gamma (solid lines) and lognormal (broken lines) distributions. Each curve shown uses the parameters that best fit the tagging data and demonstrates the different characteristic shapes of the functions. The exponential distribution gives a constant hazard rate; the gamma distribution shows an increasing hazard rate over time, and the lognormal hazard rate increases to a peak then declines.

Each term in this equation can be evaluated for any set of *x, b, d* and parameters for the distributions of animal and tag lifetimes. The lifetime of tags was assumed to be normally distributed with a high mean. Neither the mean nor the variance of tag survival was known *a priori*. We used three different distributions to model the lifetime of seals (Figure 1).

1. The simplest model used assumed that pups had a constant risk of mortality, giving rise to an exponential distribution of seal lifetimes;

2. A gamma distribution of life expectancies, which equates to an increasing risk of mortality with age;

3. A lognormal distribution, which provides a risk of mortality that increases to a peak, then declines.

The gamma and lognormal distributions are especially plausible in the present case; it is conceivable that harbour seal pup mortality involves trade-offs between the declining benefit of maternal provisioning with age and the increasing energetic demands of thermoregulation – giving rise to an increasing risk of mortality with age, and the increasing robustness of the animals as they grow larger and better able to forage independently – giving rise to a decreasing risk of mortality with age.

Twelve models were fitted to the data by maximum likelihood, with each possible pairing of the three distributions in each region. When the two regions followed the same distribution, models were fitted both with separate and shared distribution parameters. Two sets of these twelve models were tested. One set assumed that all animals were born on a common, but unknown, date to be estimated from the data; and a second set assumed a separate birth date estimated from the data for each region. Parameter optimization was performed using the ‘optim’ function in R based on the Nelder & Mead [28] method. The birth date parameter was constrained within the models such that pups could not have been born before June 1^st^ or after June 23^nd^. Using the range of neonatal weights and rates of mass gain reported in [6], [19], estimates of pup birth dates were back-calculated from their mass at tagging and tag date. The earliest birth date obtained in this manner was June 4^th^, so June 1^st^ was chosen as a conservative lower bound for both populations. The upper bound of June 23^nd^ was specified because this was the earliest tagging date. Because of the small sample sizes and relatively high numbers of parameters in the models, we used corrected Akaike information criterion (AICc) [29] for model comparison. The best approximating model was identified as the one with the lowest AICc (AICc_min_), and Akaike weights (*w_i_*) were used as estimates of the probability that each model was correct. These are based on the difference between model i's AICc score, AICc_i_, and that for the best model (Eqn 3,4) [30].
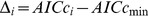
(3)

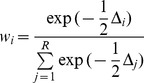
(4)


The best model was refitted to 500 bootstrap re-samples drawn from the data to calculate 95% confidence intervals of parameter estimates. In addition, to explore the potential range in survival probabilities between the two populations, we fitted each of the models where the two populations had separate distributions (18 out of 24 models) to 500 bootstrap re-samples drawn from the data. These parameter estimates were then used to calculate survival trajectories for each population and the ratio of the probability of survival to 6 months in Orkney to Lismore. 95% confidence intervals on this ratio, weighted by model AICc weights, were then used to provide a range of the potential difference in survival probability between the populations.

## Results

Transmissions were received from 48 out of 50 tags (24 from each location). All tags were operational before deployment, pups were observed swimming with their mothers after tagging, and the 48 tags produced locations soon after tagging. Therefore, the most likely reason for the two failures was considered to be attachment failure and these two individuals were excluded from data analysis.

**Figure 2:**
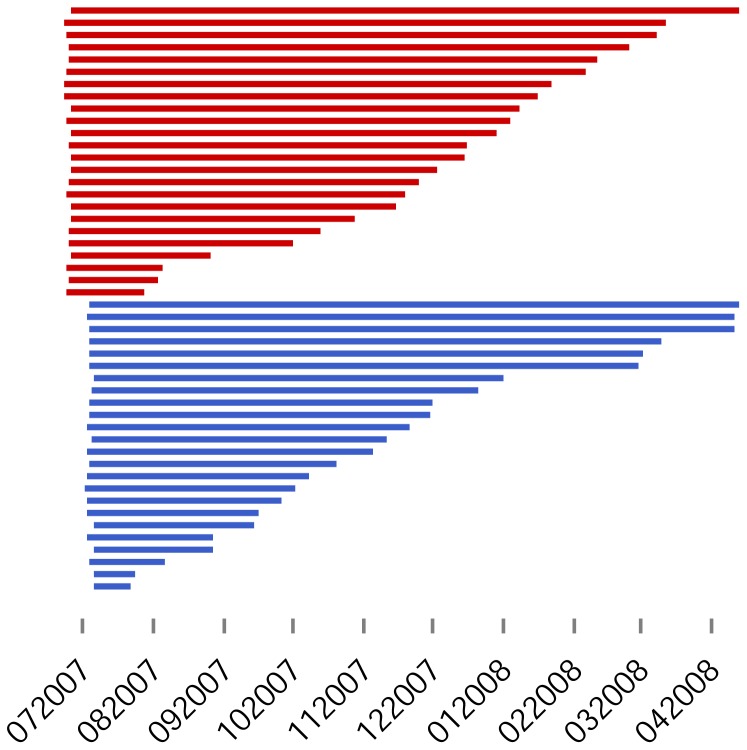
Habour seal pup tag durations. Pups were tagged on the west coast of Scotland at Lismore (red) in late June and in the Orkney Islands (blue) in early July. The date of last transmission from a live animal was determined by examining the pattern of movement from location data.

Tag durations ranged from 16 to 284 days (mean = 138.5; bootstrap 95% confidence interval on mean 104.8 – 173.2) in Orkney and 34 to 292 days (mean = 165.4; bootstrap 95% confidence interval on mean 136.1 – 194.9) in Lismore (Figure 2). The distribution of tag durations was similar between the two populations (Kolmogorov-Smirnov test; *p* = 0.26) and there were no significant differences in the mean number of transmission days (Mann-Whitney U-test; *p* = 0.19), however this test had limited power. Significant differences in mean tag duration were only obtained when 56 days were added to the Lismore tag durations.

**Figure 3:**
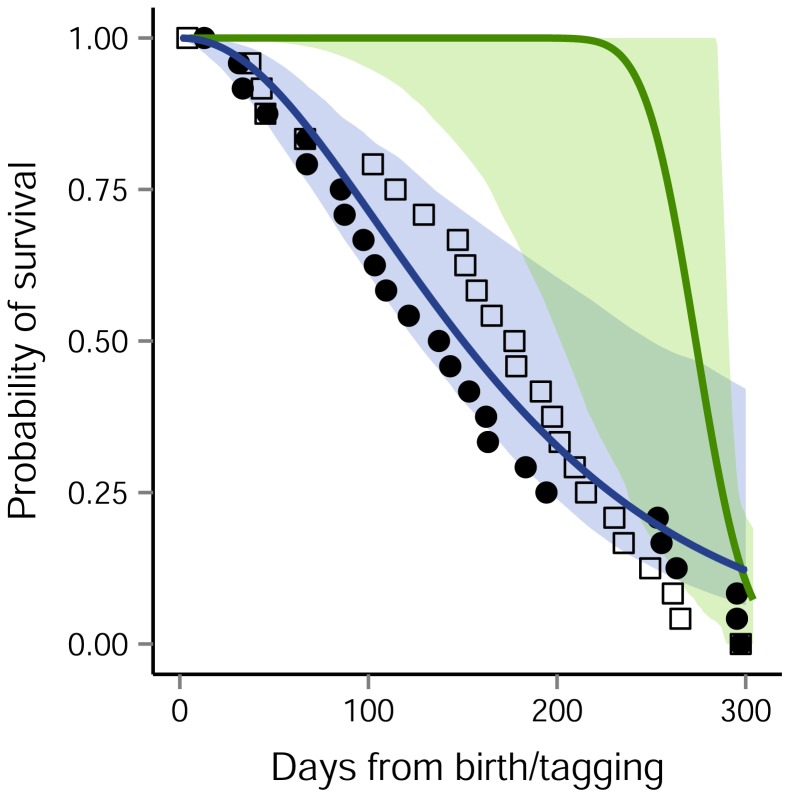
Survival of harbour seal pups and tags. Open squares are the dates of last ‘live’ transmission from tags attached to harbour seal pups at Lismore and solid circles are those deployed at Orkney. Solid lines are the estimates of pup (blue; assumed to have been born on the 20^th^ June) and tag (green; assumed to have been attached on 23^rd^ June) survival from the best of the fitted models, which had a common gamma distribution for survival for both Orkney and Lismore and a common birth date. Shaded regions show 95% confidence limits generated from 500 bootstrap re-samples of the data.

The model with the lowest AICc value and highest Akaike weight assumed both populations followed a common gamma distribution and shared a single birth date estimated from the data (gamma; Single; w = 38%; Table 2). The empirical support for this model was 5.5 times stronger than the best model which estimated separate distribution parameters for each population (lognormal-gamma; Single) but only 1.6 times stronger than the model which assumed both populations followed a common lognormal distribution. Most model weights (68%) were on models with shared distribution parameters for pup survival and 57% also had a common birth date parameter, suggesting that both pup birth date and the probability of pup survival were similar in the Orkney and Lismore populations. The model which best approximated the data returned a gamma distribution for pup mortality with rate 0.015 (95% CI 0.005 – 0.022) and shape 2.57 (95% CI 1.42 – 3.72) parameters, and tag survival parameters μ = 270 (95% CI 197 – 287) days and σ = 21 (95% CI 1 – 67) days (Table 3). The estimate of the birth date (assumed to be constant for all individuals) was 20 June, 2007 (95% CI 14 June – 23 June). Pup and tag survival curves are plotted in Figure 3. Pup survival probability from this model was high (approximately 0.90), for the first ∼50 days, then declined at a roughly constant rate. Pup survival to six months was 0.390 (95% CI 0.297 – 0.648) from this model. Tag survival was high and constant until around day 250 when tag failure probability increased rapidly (Figure 3).

**Table 2:** Corrected Akaike Information Criterion and weights for the candidate models.

		AICc		ΔAICc^a^		*w* b		
Orkney	Lismore	Single^c^	Separate^c^	Single	Separate	Single	Separate	Total
gamma		556.99	560.05	0.00	3.06	0.31	0.07	0.38
lognormal		557.94	561.19	0.95	4.20	0.19	0.04	0.23
lognormal	gamma	560.39	563.07	3.40	6.08	0.06	0.01	0.07
exponential		560.63	563.12	3.63	6.13	0.05	0.01	0.07
exponential	gamma	560.71	563.45	3.72	6.46	0.05	0.01	0.06
gamma	gamma	561.12	563.93	4.13	6.94	0.04	0.01	0.05
exponential	exponential	561.27	563.89	4.28	6.90	0.04	0.01	0.05
lognormal	exponential	562.34	564.70	5.35	7.71	0.02	0.01	0.03
exponential	lognormal	562.62	565.35	5.63	8.36	0.02	0.00	0.02
gamma	lognormal	563.23	565.57	6.24	8.58	0.01	0.00	0.02
lognormal	lognormal	563.24	564.94	6.25	7.95	0.01	0.01	0.02
gamma	exponential	566.89	565.95	9.90	8.96	0.00	0.00	0.01

a the difference between AICc_i_ and AICc_min_. See Eqn (3) in text.

b Akaike weights. See Eqn (4) in text for calculation.

c Single  =  single birth date parameter estimated for both populations; Separate  =  separate birth date parameter estimated for each population

**Table 3:** Parameter estimates for the best model.

Process	Parameter	Mean estimate	95% CI
	Birth date	20 June	14 June – 23 June
Pup survival	rate	0.015	0.005 – 0.022
	shape	2.570	1.42 – 3.72
Tag survival	mean	270	197 – 287
	SD	21	1 – 67

The confidence intervals are generated from 500 bootstrap re-samples of the data.

Models (n = 18) where two separate distributions were included for Orkney and Lismore represented 32% of total model AIC weights. To explore the potential uncertainty in survival probabilities between the two populations, we calculated the model averaged ratio of probability of survival to 6 months (*S(t = 180 days)*), weighted by the AICc weights [30], from 500 bootstrapped estimates of distribution parameters. A ratio of 1 indicates that there is no difference in *S(t = 180 days)* between Orkney and Lismore; ratios ranged from 0.63 – 1.55 (95% CI).

**Figure 4:**
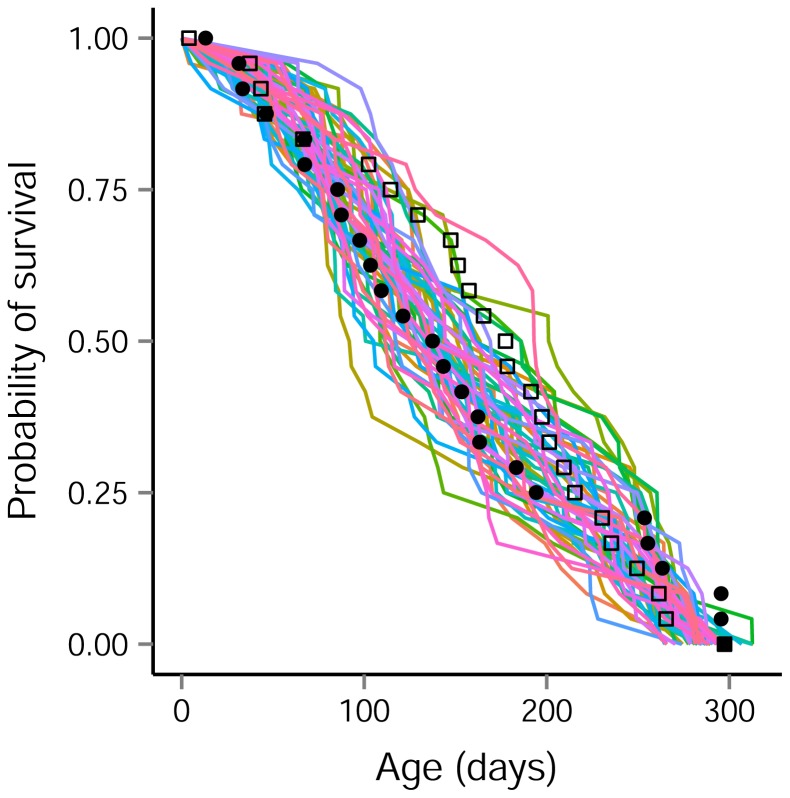
Survival trajectories of simulated tagged pups. Each line connects the last ‘live’ transmission day for a set of 24 simulated tagged harbour seal pups (100 simulations). Pup mortalities were drawn from a gamma distribution. Animals were considered to be tagged at birth and tag failure to be normally distributed. All parameter values were taken from Table 2. The Orkney (solid circles) and Lismore (open squares) data also are shown.

In models where the two populations shared distribution parameters – such as the model of best fit – the above method assumes that observations are independent from one another; however, this assumption may be unrealistic and in such cases, there is a risk of model overfitting. To explore this possibility, we simulated 100 random survival probability trajectories for 24 pups generated using the distribution parameters for pup mortality and tag failure from the best-fitting model (gamma; Common). The observed pattern of mortality for both Orkney and Lismore pups fell within the spread of the simulated trajectories, suggesting that the fitted model had adequate predictive performance (Figure 4).

## Discussion

The aim of the present analysis was to determine whether the rapid decline in the number of harbour seals in Orkney could be due to an increase in pup mortality. This is the first study of its kind to use satellite transmitters deployed on a pre-weaned pinniped to estimate pup survival. We present two clear pieces of evidence that additional mortality among young pups is not the primary factor driving the population decline in Orkney: we show that the mortality schedule in Orkney was broadly similar to that in another population where growth was stable, and that at least 29% of harbour seal pups could be expected to survive their first six months.

### Pup survival and population decline

The validity of our results relied in large part on the validity of the assumptions underlying the statistical approach. Specifically, we assumed that:

1. Individuals sampled were representative of the population;

2. In models with shared distribution parameters (6 out of 24 models), ‘system failure’ events were independent from one another;

3. The process of tagging did not affect individual survival.

Harbour seal pups caught for tag deployment were targeted on the basis of sex. Sex-specific differences in pup mortality rate have been reported for other phocid species [24], [31] and it is possible that there are differences in male pup survival between Orkney and Lismore. The present study focused on female harbour seal pups because we were most interested in those parameters likely to have the greatest influence on the rate of population decrease. By focusing our sampling on this subset of the population, we increase the robustness of our inferences about female harbour seal pup mortality. As is often the case for free-ranging wild animals, assumption (2) potentially was not met; here because of regional clustering in the data. Pups from a particular colony could be exposed to similar mortality agents [32]. As presented in the results, there is a danger of model overfitting in such cases; however, repeated simulations of new data using the parameters from the best fitting model produce repeatable and typical patterns of mortality, suggesting model overfitting was not a concern. There was no way to assess directly whether or not assumption (3) was met. However, all tags transmitted for at least 34 days from the date of tagging in Lismore and 16 days from the date of tagging in Orkney. Assuming that pups were born around the 20^th^ of June, the youngest pup stopped transmitting at 32 days old which was likely to be some time after weaning (approximately 24–28 days after birth [6], [19]). Small pups would not survive for more than a few days without suckling so this was compelling evidence that the tagging did not unduly affect the mother pup bond in any instance.

Both the lognormal and gamma distributions for pup mortality were preferred to a simple exponential, with the gamma returning the best fitting model. This is consistent with pups experiencing lower mortality during the early post weaning period, when they may have some energetic buffer from recent maternal investment. Mortality rate then increases through the year as pups forage independently, encounter their first winter and must cope with the increased energetic demands of thermoregulation [23]. Early winter may also be a time of increased competition with newly weaned grey seal pups.

Post-weaning pup mortality and survival to year one can be extremely variable in phocid seals as they adapt to their environment and develop the physiological and behavioural requirements for diving and foraging. In an increasing population of northern elephant seals, the probability of surviving to year one was 0.368±8.5 (*n* = 8,362) [33] and 0.459±0.091 (n = 3,606) in a declining population of female southern elephant seals on Macquarie Island [31] Hall et al. [24] found large differences between survival probabilities for male and female grey seal pups off the east coast of Scotland. Annual survival rate for females was 0.617±0.155 (*n* = 108), but dropped to 0.193±0.084 (*n* = 96) for males. For harbour seals on the Swedish west coast, Harding et al. [23] found that pup survival was highly dependent on their autumn mass, with individual survival probabilities ranging from 0.4 for small pups to over 0.9 for large pups. The best model in our analysis estimated female pup survival probability to be 0.390 (95% CI 0.297 – 0.648) to six months, and did not distinguish a separate rate for each population, despite the fact that the population at Lismore has been stable over the past decade while the Orkney population continues to decline. This value is low, but falls within the range reported in the literature for both increasing and decreasing populations of phocid seals. Importantly, our model is a composite of survival and censoring processes; it is possible that early failure of telemetry devices was attributed to early pup death, lowering the estimated pup survival probabilities. Additionally, the ratio test indicated that survival probabilities potentially could be different between the two populations (95% CI on the ratio of Orkney:Lismore survival to six months was 0.65 – 1.55). If the true ratio was near the lower end of this interval, increased pup mortality in the Orkney population could be a contributory factor in its decline.

The most recent estimate of the population decline at Orkney is 13% per annum (95% CI 10.8 – 14.8) [11]. Our results suggest that approximately 40% of pups survived to 6 months and the model predicted lower survival for pups over the winter to day 300. This pattern is consistent across both populations but our data provided limited scope for assessing pup survival to year one. Predicted survival to 300 days was 0.12 (95% CI 0.064 – 0.42). Even if there was a complete failure in juvenile recruitment at Orkney, an 11% *per annum* decline could only be produced if adult survival was below 89%. If all females reproduce annually from age 4 onwards [34], half of pups are female and 42% of these survive their first year, then a simple deterministic model that results in exponential growth or decline requires annual survival, beyond the first year, to be 0.82 to give an 11% per annum rate of decline. Presently, there are no age-structured models of harbour seal population dynamics in the UK. Nor are there estimates of adult survival rates for either the Orkney or Lismore populations. Mackey et al. [35] estimated a 0.97 (95% CI 0.92 – 0.99) survival probability for adult harbour seals in the Cromarty Firth, in north-east Scotland, from photo identification mark-recapture of live animals. The authors note that their estimate is high compared to others reported in the literature [34], but corresponds well to a similar study in a nearby population which found female survival to be 0.95 (95% CI 0.91 – 0.97; Line Cordes pers comm.). Further study is required on the Orkney population to ascertain whether adult survival could be low enough to account for the reduction in harbour seal numbers. Aside from an increase in adult mortality, a substantial reduction in female reproductive rates could be driving the decline. At present, there is no information available on fecundity for either population, though the availability of pups for tagging in this study does indicate that there cannot have been total reproductive failure at Orkney.

### Birth date

The values estimated for the birth dates (Table 3) need to be treated with caution as they act as an offset to the start of mortality. However, the estimate of the birth date assumed to be constant for all individuals (20^th^ June, 2007) is plausible. Reported neonate birth weights at other harbour seal colonies in the North Atlantic range from ∼8 to11kg [6], [34]. Lismore pups had a mean weight at capture of 11.5kg. This and the fact that many of these pups still had visible remnants of the umbilicus suggests these pups were only a few days old when tagged between the 23^rd^ and 26^th^ of June, 2007. Orkney pups were around 60% heavier (18.5kg) at their capture 10–12 days later. This represents an approximate growth rate of 0.58 to 0.7 kg*d^−1^ similar to estimates from Sable Island and the Gulf of St Lawrence [6], [19], suggesting that the Orkney pups were indeed older and likely to have been born at a similar time to the Lismore pups. The rejection of separate birth dates for the two regions may be more meaningful than the absolute date estimated from the model, especially considering that the Akaike weights of models having a common birth date totaled 0.8. Because we had no information on the age of individuals at tagging, nor on typical neonatal survival rates for these populations, it is perhaps unsurprising that the models converged on a common birth date. Little information was present with which to determine differences in birth dates between the two populations or indeed between individuals.

### Survival from telemetry data

Obtaining estimates of pup survival from satellite telemetry data presented a challenge because of the uncertainty in differentiating between animal mortality and tag failure. Marine mammal deaths usually only are observed if the animal is washed ashore or if it is killed intentionally – neither of which provide unbiased data on natural mortality. Pup death was not observed directly in the present study. Instead, we used the tagging date and the last date of transmission from a moving tag to model the distribution of pup mortality and tag failure. The epoxy attachment method used in 2007 was highly effective for adults and meant it was much more likely that tag batteries or aerials would fail before accidental detachment occurred. Importantly, the attachment procedures were identical for both populations and the probability of tag failure should be the same. The rapid decline in tag survival after ∼8 months (Figure 3) is consistent with this assumption, but the wide range in 95% confidence intervals for tag failure demonstrate the uncertainty surrounding this parameter after ∼5 months (150 days).

The problem of tag failure in telemetry studies, leading to ‘right-censored data’, is not new. Some methods circumvent the problem by empirical estimation of tag failure probabilities [36], while others propose statistical methods to incorporate these data into survival probability estimates [7]. However, many approaches either omit right-censored individuals from the analysis, or make strong assumptions about them which can limit the scope of inference on age-specific survival [37]. Bayesian approaches to modelling survival data such as BaSTA (Bayesian Survival Trajectory Analysis) can account for both left-truncated and right-censored individuals in a Bayesian hierarchical framework [37], [38], and maximum likelihood. While such models are appealing for decomposing lifetime mortality schedules, the present data spanned only a fraction of harbour seal life history and represented survivorship for the first hazard only, plus tag failure – or tag senescence. Importantly, our dataset did not include any animals of known age-at-death and all individuals in the study had to be considered right-censored and left-truncated. Animal age at entry to the study was unknown and animal death was never directly observed and could only be inferred from the pattern of movement in tag transmissions. Because we expected tag senescence to occur at some point during the study period, and to censor pup survival after a reasonable amount of time, we modelled separate parameters for tag failure, assuming a normal distribution and high mean of tag durations. As far as the authors are aware, there are no empirical data on the likely distribution of tag failure probabilities. While tags can fail for a variety of reasons, we reasoned that the most likely cause of tag failure in the present study was battery exhaustion which was expected to occur around February. A Gaussian distribution was therefore used to represent tag failure. While traditional approaches to modelling survival from capture-recapture/recovery data could have been applied in the present study, they would require additional assumptions about consistency in the sightability of the animals over a period when their behaviour develops and changes substantially.

### Conclusions and implications

We conclude that an increase in pup mortality is not the primary driver of the decline seen in abundance of Orkney harbour seals over the past decade, although it could be a contributory factor. The present study is limited in scope to a single year and to comparison with only one other stable population. In reality, population demographic parameters can change significantly between years, and between populations. The low study power constrains the inferences that can be made about pup survival probabilities but the long-term mark-recapture studies necessary for detailed examination of population dynamics [6], [23] simply are not feasible. In their absence, we have shown that an estimate of pup survival can be obtained from ‘short-term’ telemetry data at relatively low disturbance cost to the focal populations. The important conclusion that pup survival in a rapidly declining population is similar to that of a stable population will help to focus hypotheses about the drivers of the decline. The concurrent declines evident in some other British populations [10] indicate that the problem is not confined to Orkney and may implicate regional-scale impacts on UK harbour seals. The OSPAR international convention (www.ospar.org) includes among its measures of Ecological Quality a requirement that North Sea harbour seal populations should not decline by more than a total of 10% over a five-year period [4]. This requirement clearly has not been met for Orkney and management action is necessary and requires further investigation of the proximate, and ultimate, causes for population decline. We suggest that such work would best concentrate at examining effects on the survival of adult harbour seals.

## Supporting Information

Appendix S1 Dataset.
(CSV)

Appendix S2 Data analysis.
(ZIP)

## References

[1] Lonergan M (2011) Potential biological removal and other currently used management rules for marine mammal populations: A comparison Marine Policy. 35: 584–589.

[2] Collen B, Loh J, Whitmee S, McRae L, Amin R, et al (2008) Monitoring change in vertebrate abundance: the Living Planet Index. Conservation Biology 23: 317–327. 19040654 10.1111/j.1523-1739.2008.01117.x

[3] Boyd IL, Wanless S, Camphuysen CJ (2006) Top Predators in Marine Ecosystems: Their Role in Monitoring and Management. Cambridge: Cambridge University Press. 378 p.

[4] OSPAR (2009) The OSPAR system of Ecological Quality Objective for the North Sea, a contribution to OSPAR's Quality Status Report 2010. Available at: http://www.ospar.org/documents/dbase/publications/p00404_working%20for%20a%20health%20north%20sea_abr.pdf.

[5] Barlow J, Boveng P (1991) Modling age-specific mortality for marine mammal populations. Marine Mammal Science 7: 50–65.

[6] Bowen WD, Ellis SL, Iverson SJ, Boness DJ (2003) Maternal and newborn life-history traits during periods of contrasting population trends: implications for explaining the decline of harbour seals (*Phoca vitulina*), on Sable Island. Journal of Zoology 261: 155–163.

[7] Fieberg J, DelGiudice GD (2011) Estimating age-specific hazards from wildlife telemetry data. Environmental and Ecological Statistics 18: 209–222.

[8] Thompson D, Duck CD, Lonergan ME (2010) The status of harbour seals (*Phoca vitulina*)in the United Kingdom. NAMMCO Scientific Publications 8: 117–128.

[9] Härkönen T, Dietz R, Reijnders P, Teilmann J, Harding K, et al (2006) A review of the 1988 and 2002 phocine distemper virus epidemics in European harbour seals. Diseases of Aquatic Organisms 68: 115–130. 16532603 10.3354/dao068115

[10] Lonergan M, Duck CD, Thompson D, Mackey BL, Cunningham L, et al (2007) Using sparse survey data to investigate the declining abundance of British harbour seals. Journal of Zoology 271: 261–269.

[11] Lonergan M, Duck C, Moss S, Morris C, Thompson D (2013) Rescaling of aerial survey data with information from small numbers of telemetry tags to estimate the size of a declining harbour seal population. Aquatic Conservation: Marine and Freshwater Ecosystems 23: 135–144.

[12] Härkönen T, Harding K, Rasmussen TD, Teilmann J, Dietz R (2007) Age- and sex-specific mortality patterns in an emerging wildlife epidemic: the phocine distemper in european harbour seals. PLoS ONE: e887. 10.1371/journal.pone.0000887 PMC1964516 17849016

[13] Jansen JK, Boveng PL, Dahle SP, Bengtson JL (2010) Reaction of harbour seals to cruise ships. Journal of Wildlife Management 74: 1186–1194.

[14] Dietz R, Teilmann J, Andersen SM, Rigét F, Olsen MT (2012) Movements and site fidelity of harbour seals (*Phoca vitulina*) in Kattegat, Denmark, with implications for the epidemiology of the phocine distemper virus. ICES Journal of Marine Science 69: 1–10.

[15] Cunningham L, Baxter JM, Boyd IL, Duck CD, Lonergan M, et al (2009) Harbour seal movements and haul-out patterns: implications for monitoring and management. Aquatic Conservation: Marine and Freshwater Ecosystems 19: 398–407.

[16] SCOS (2011) Scientific Advice on Matters Related to the Management of Seal Populations: 2011. Sea Mammal Research Unit, University of St Andrews. Available at: http://www.smru.st-andrews.ac.uk/documents/678.pdf.

[17] Eberhardt LL (1985) Assessing the dynamics of wild populations. The Journal of Wildlife Management 49: 997–1012.

[18] Siler W (1979) A competing-risk model for animal mortality. Ecology 60: 750–757.

[19] Dubé Y, Hammill MO, Barrette C (2003) Pup development and timing of pupping in harbour seals (*Phoca vitulina*) in the St. Lawrence River estuary, Canada. Canadian Journal of Zoology 81: 188–194.

[20] Jørgensen C, Lydersen C, Brix O, Kovacs KM (2001) Diving development in nursing harbour seal pups. The Journal of Experimental Biology 204: 3993–4004. 11807117 10.1242/jeb.204.22.3993

[21] Greaves DK, Schreer JF, Hammill MO, Burns JM (2005) Diving heart hate development in postnatal harbour seals, *Phoca vitulina* . Physiological and Biochemical Zoology 78: 9–17. 15702458 10.1086/425201

[22] Prewitt JS, Freistroffer DV, Schreer JF, Hammill MO, Burns JM (2010) Postnatal development of muscle biochemistry in nursing harbor seal (*Phoca vitulina*) pups: limitations to diving behavior? Journal of Comparative Physiology B 180: 757–766. 10.1007/s00360-010-0448-z 20140678

[23] Harding KC, Fujiwara M, Axberg Y, Härkönen T (2005) Mass-dependent energetics and survival in harbour seal pups. Functional Ecology 19: 129–135.

[24] Hall AJ, McConnell BJM, Barker RJ (2001) Factors affecting first-year survival in grey seals and their implications for life history strategy. Journal of Applied Ecology 70: 138–149.

[25] Bowen WD, Iverson SJ, Boness DJ, Oftedal OT (2001) Foraging effort, food intake and lactation performance depend on maternal mass in a small phocid seal. Functional Ecology 15: 325–334.

[26] Hays GC, Bradshaw CJA, James MC, Lovell P, Sims DW (2007) Why do Argos satellite tags deployed on marine animals stop transmitting? Journal of Experimental Marine Biology and Ecology 349: 52–60.

[27] R Core Development Team (2011) R: A language and environment for statistical computing. Vienna: R Foundation for Statistical Computing.

[28] Nelder JA, Mead R (1965) A simplex method for function minimization. The Computer Journal 7: 308–313.

[29] Hurvich CM, Tsai C-L (1989) Regression and time series model selection in small samples. Biometrika 76: 297–307.

[30] Burnham KP, Anderson DR (2002) Model Selection and Multi-Model Inference. New York: Springer.

[31] Hindell MA (1991) Some life-history parameters of a declining population of southern elephant seals, *Mirounga leonina* . Journal of Animal Ecology 60: 119–134.

[32] Murray D (2006) On improving telemetry-based survival estimation. The Journal of Wildlife Management 70: 1530–1543.

[33] Le Boeuf BJ, Morris P, Reiter J (1994) Juvenile survivorship of northern elephant seals. Elephant Seals: Population Ecology, Behavior and Physiology. Berkeley: University of California Press. pp. 121–136.

[34] Härkönen T, Heide-Jørgensen M-P (1990) Comparative life histories of east Atlantic and other harbour seal populations. Ophelia 32: 211–235.

[35] Mackey BL, Durban JW, Middlemas SJ, Thompson PM (2008) A Bayesian estimate of harbour seal survival using sparse photo-identification data. Journal of Zoology 274: 18–27.

[36] Holbrook CM, Perry RW, Brandes PL, Adams NS (2013) Adjusting survival estimates for premature transmitter failure: a case study from the Sacramento-San Joaquin Delta. Environmental Biology of Fishes 96: 165–173.

[37] Colchero F, Clark JS (2011) Bayesian inference on age-specific survival for censored and truncated data. Journal of Animal Ecology 81: 139–149. 21883202 10.1111/j.1365-2656.2011.01898.x

[38] Colchero F, Jones OR, Rebke M (2012) BaSTA: an R package for Bayesian estimation of age-specific survival from incomplete mark–recapture/recovery data with covariates. Methods in Ecology and Evolution 3: 466–470.

